# Tumors associated with radiotherapy: a case series

**DOI:** 10.1186/s13256-020-02482-x

**Published:** 2020-10-06

**Authors:** Mauricio Garcia, Dary L. Hernandez, Sara Mendoza, Nelson Buelvas, Angelina Alvarez, Jose Esguerra, Sandra Díaz

**Affiliations:** 1 Oncologic Surgery Service, National Cancer Institute, Street 1 # 9-85, Bogota, Colombia; 2 Breast and Soft Tissue Surgery Service, National Cancer Institute, Bogota, Colombia; 3 Radiotherapy Oncology Service, National Cancer Institute, Bogota, Colombia

**Keywords:** Breast cancer, Breast-conserving therapy, Postirradiation, Radiotherapy

## Abstract

**Background:**

Breast cancer is the cancer with the highest incidence and mortality worldwide. Its treatment is multidisciplinary with surgery, systemic therapy, and radiotherapy. In Colombia, according to Globocan 2018, there is an age-standardized incidence rate of 44 per 100,000 women. Radiotherapy improves local and regional control in patients with breast cancer, and it could even improve relapse-free survival and overall survival in patients with nodal disease. The toxicity of this treatment in most cases is mild and transient, but in a low percentage of patients, radiotherapy-induced tumors may develop.

**Case presentation:**

Seven Colombian patients treated for breast cancer at our institution developed radiotherapy-induced tumors between 2008 and 2018. The median age was 54.4 (range 35–72) years. Six patients had locally advanced tumors at the time breast cancer was diagnosed, and all of them received neoadjuvant or adjuvant chemotherapy and radiotherapy. The radiotherapy-induced tumors were five sarcomas, one of which was a well-differentiated angiosarcomatous vascular lesion with negative c-Myc (benign lesion), and the remaining patient had basal cell carcinoma associated with radiotherapy.

**Conclusions:**

Sarcomas are the most common radiotherapy-induced tumors after breast cancer treatment. These are rare, aggressive tumors and represent between 0.5% and 5.5% of all sarcomas. Basal cell carcinoma has also been associated with breast cancer treatment. The management is individualized and multimodal, including surgical resection and chemotherapy. Different studies have shown that radiation therapy is a risk factor for the development of soft tissue tumors.

## Introduction

Breast cancer is the cancer with the highest incidence and mortality for women worldwide. A total of 2,088,849 new cases were reported in 2018, corresponding to 11.6% of all cancer cases, and 626,679 died of this disease, corresponding to 6.6% of all cancer deaths [1].

Breast cancer treatment requires a multidisciplinary approach that includes the possibility of surgical resection, radiotherapy, and systemic treatments such as chemotherapy, targeted therapies, and hormone therapies [2]. Among the long-term adverse effects reported in the literature is the appearance of sarcomas, which can be associated with chronic lymphedema (Stewart-Treves syndrome) or radiation exposure; the latter are called “radiotherapy-induced sarcomas” [3].

It has been specified that radiotherapy increases the risk of skin, lung, thyroid, esophageal, and soft tissue tumors (5-year recurrence rate 2.53). Among radiotherapy-induced tumors, sarcomas are the mainly described histology [4]. In the U.S. Surveillance, Epidemiology, and End Results Program (SEER) registry, of 563,155 women diagnosed with breast cancer between 1973 and 2003, 211,027 were treated with radiotherapy; 0.07% of these women developed soft tissue sarcomas in the breast or ipsilateral arm, and the 5-year overall survival rate was 38% [5]. Similarly, literature reports the association of radiotherapy exposure with the development of benign pathologies such as vascular skin lesions, which can eventually present malignant transformation to angiosarcoma [6].

In 1948, Cahan *et al.* defined four criteria, subsequently modified by Arlen *et al*., for the diagnosis of sarcomas associated with radiotherapy: (1) sarcoma must appear within previously irradiated fields; (2) there should be no evidence that sarcoma was present before the onset of radiation; (3) it must have a different histology from that of the primary condition confirmed by biopsy; and (4) the onset of sarcoma must occur at least 6 months after ending the radiation therapy [3, 7]. Currently, the latency for the possible appearance of these tumors is not well established, hence the importance of timely follow-up. The most common histological subtypes of radiotherapy-induced sarcomas are undifferentiated pleomorphic sarcoma, angiosarcoma, osteosarcoma, and fibrosarcoma [8].

The National Cancer Institute in Bogota, Colombia (a national reference center), evaluates approximately 685 patients with breast cancer each year, of whom about 80% receive radiotherapy either because they have locally advanced tumors or after conservative surgery. Seven cases of patients who developed second tumors associated with radiotherapy from 2008 to 2018 with a previous breast cancer are presented in this report.

## Case presentation

The median age of the Colombian patients was 54.4 (range 35–72) years at the time of breast cancer diagnosis. Six patients presented with locally advanced tumors at the time of their first diagnosis (three with stage IIB, two with stage IIIB, and one with stage IIIA). The remaining patient was classified as stage IIA.

Histopathologically, five patients had luminal A tumors, one had a *BRCA1* mutation, one had a luminal tumor that could not be stablished as A or B because Ki67 was not available, and one had triple-negative disease (Table 1). All patients received chemotherapy regimens that consisted of anthracyclines and taxanes, either neoadjuvant or adjuvant. In relation to surgical treatment, conservative surgery of the breast was performed in four cases and modified radical mastectomy in three cases; adjuvant hormone therapy was given to patients positive for hormone receptors, and adjuvant radiotherapy was completed in all patients, performed with a three-dimensional conformational technique, 2-Gy fractioning, and a complete dose from 50 to 60 Gy. The detailed characteristics of patients in relation to their primary breast tumor, treatment received, and diagnosis of the lesion associated with the corresponding radiotherapy are summarized in Table 1.

**Table 1:** Characteristics of the primary tumor

Characteristics	Number (%)
Age, years (median)	54.4 (35–72)
Breast cancer clinical stage	
IIA	1 (14.3%)
IIB	3 (42.8%)
IIIA	1 (14.3%)
IIIB	2 (28.6%)
Tumor biology	
Luminal A	6 (85.7%)
Triple-negative	1 (14.3%)
Primary tumor treatment	
Neoadjuvant chemotherapy	1 (14.3%)
Adjuvant chemotherapy	2 (28.6%)
Neoadjuvant + adjuvant chemotherapy	4 (57.1%)
Radical surgery	3 (42.8%)
Conservative surgery	4 (57.1%)
Adjuvant radiotherapy	7 (100%)
Hormone therapy	6 (85.7%)

In our series, the time elapsed between diagnosis of the primary tumor and diagnosis of the tumor associated with radiotherapy varied between 5 years and 10 years, with a mean of 6.7 years. Histopathology following biopsies revealed five cases as sarcomas, one angiosarcoma (very well differentiated, c-Myc negative (benign lesion), and one basal cell carcinoma associated with radiotherapy, which appeared as two lesions with different onset periods, one at 6 years and another at 14 years from the first diagnosis.

**Fig. 1:**
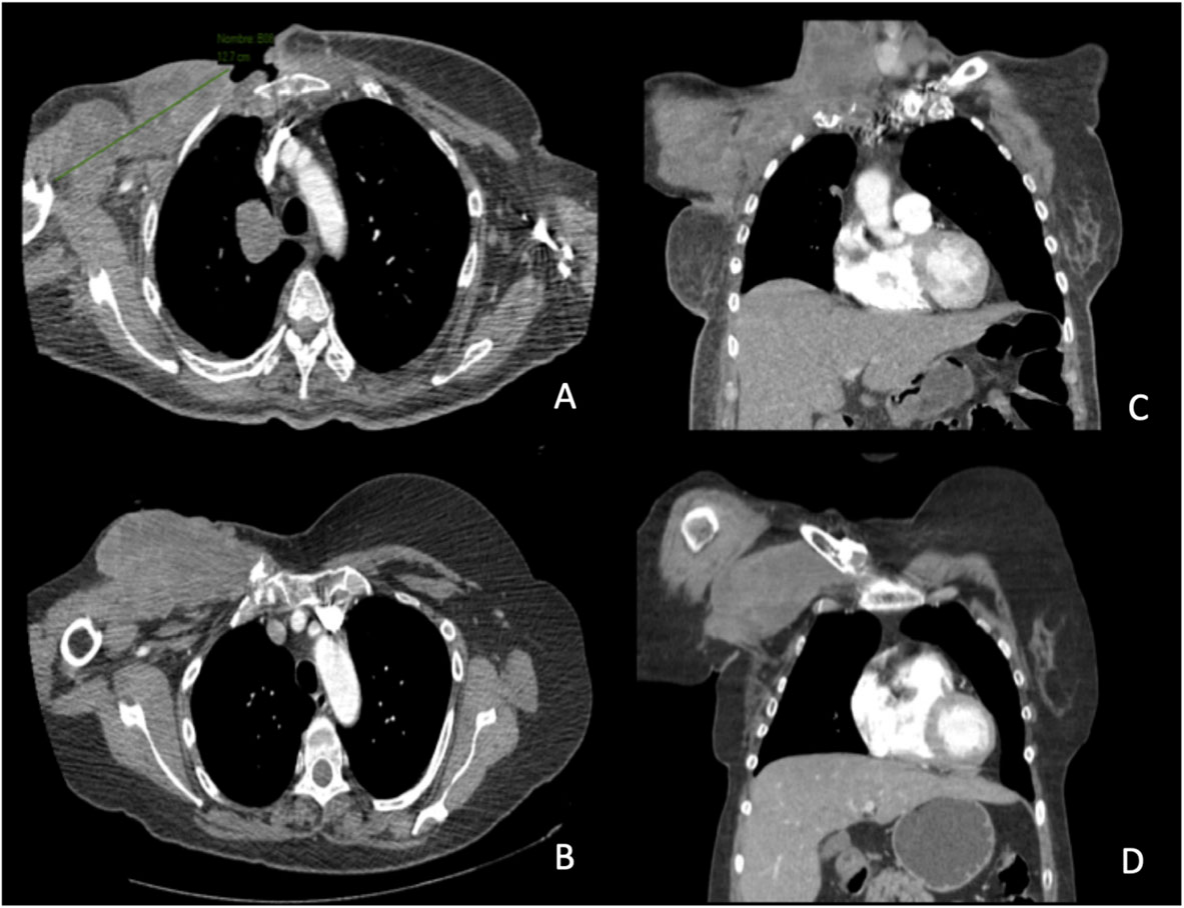
Thoracic computed tomography in axial (**a**, **b**) and coronal (**c**, **d**) projections of two of the patients, showing an infraclavicular mass appearance on the thoracic wall and axillary extension, all coinciding with the radiation fields

**Fig. 2:**
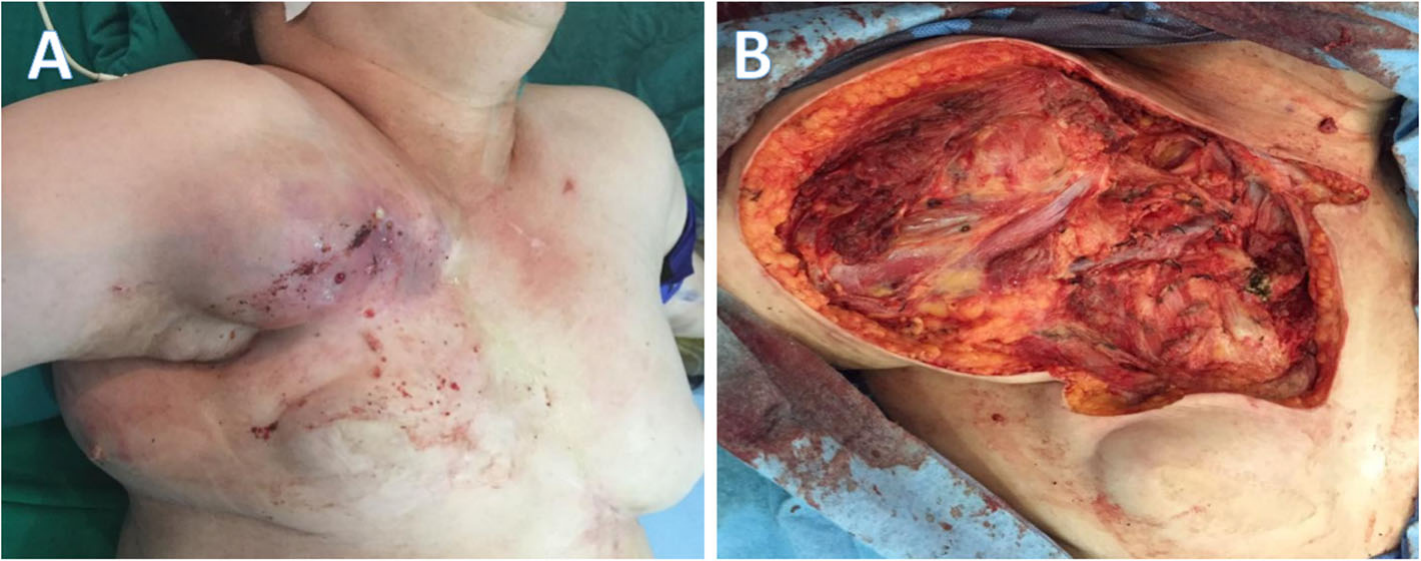
**a** Preoperative aspect of a sarcoma associated with radiotherapy that causes local inflammatory changes. **b** Intraoperative imaging after sarcoma resection including clavicle resection

Regarding histology of the sarcomas, two corresponded to pleomorphic sarcomas with osteoid component, two to high-grade angiosarcomas, and the remainder was a high-grade carcinosarcoma. All secondary tumors were located in the previous radiotherapy field (Fig. 1), appearing more frequently in the breast (42.8%), followed by the chest wall (28.6%) or sternum and infraclavicular in 14.3%, respectively (Table 2). Many of these tumors were large, averaging 10 cm in diameter, conditioning an exophytic component tending to skin ulceration (Fig. 2).

**Table 2:** Characteristics of the second primary malignancies

Characteristic	N (%)
Time of onset	
5 years	2 (28.6%)
6–9 years	4 (57.1%)
> 10 years	1 (14.3%)
Location	
Breast	3 (42.8%)
Sternum	1 (14.3%)
Infraclavicular	1 (14.3%)
Chest wall	2 (28.6%)
Histology	
Pleomorphic fusocellular sarcoma with osteochondromatous component	2 (28.6%)
High-grade epithelioid angiosarcoma	2 (28.6%)
Carcinosarcoma	1 (14.3%)
Well-differentiated angiosarcoma vascular lesion c-Myc (−)	1 (14.3%)
Nodular basal cell carcinoma	1 (14.3%)

Once the tumor diagnoses were confirmed, all patients were presented to multidisciplinary tumor boards, in which the services of breast and soft tissue surgery, clinical oncology, radiation oncology, oncology pathology, oncological radiology, and oncological dermatology participated. The therapeutic decisions to be followed with each patient were defined in these meetings. The five patients diagnosed with sarcomas were taken to wide local resection with margins of 3 cm (Fig. 2). The patient who developed the well-differentiated vascular lesion was handled with clinical follow-up, and the patient with basal cell carcinomas was treated with cryosurgery.

Two patients were offered adjuvant chemotherapy with mesna, anthracycline, and ifosfamide. One of them rejected chemotherapy at first but received anthracycline and platinum when the disease progressed.

Two patients received adjuvant radiotherapy with three-dimensional conformal technique, 1.8-Gy fractions, and a total dose of 30 and 45 Gy. These doses were based on the fields to be irradiated, the organs at risk, and accumulated toxicity of the previous radiotherapy.

Progressive disease was seen in four of five cases in lung, pleura, chest wall, and regional nodes, and the patients died between 12 and 38 months after diagnosis. Only one of the five patients diagnosed with sarcoma is alive without disease, as are the two patients diagnosed with benign vascular lesion and basal cell carcinoma (Table 3).

**Table 3:** Treatment of the second primary malignancies

Surgical treatment	
Wide local resection + partial cleidectomy	1 (14.3%)
Wide local resection + chest wall resection	1 (14.3%)
Wide local resection + cleidectomy + sternotomy + chest wall resection	1 (14.3%)
Simple mastectomy	1 (14.3%)
Radical mastectomy	1 (14.3%)
Cryosurgery	1 (14.3%)
Adjuvant treatment	
Chemotherapy	2 (28.6%)
Radiotherapy	2 (28.6%)
Did not receive	3 (42.8%)
Not applicable	1 (14.3%)
Outcome	
Death due to disease	4 (57.2%)
Alive and disease-free	3 (42.8%)

## Discussion

Breast cancer is the cancer entity most frequently diagnosed in women. The overall survival rate in patients with early breast cancer has increased over the years, largely as a result of adjuvant systemic treatment (chemotherapy, hormone therapy, and targeted therapies) [1]. In breast cancer, radiation therapy is indicated in patients with locally advanced disease and in patients who undergo conservative breast surgery. This treatment modality has been able to demonstrate benefits in relapse-free survival and overall survival [2]. However, its use has been described as an established risk factor for the development of other malignant tumors. Some sites show a significantly higher relative risk of developing tumors associated with radiotherapy, such as the thyroid, esophagus, lung, and soft tissues. This risk depends on the technologies used, the area treated, the equivalent biological dose, the dose per fraction, fractionation, the duration of treatment, bone marrow exposure, and personal sensitivity [9].

Within the genomics of tumors, implication of the rearrangement of the *RET* proto-oncogene in thyroid cancer and amplification of MYC in cutaneous angiosarcoma after irradiation of the chest wall for breast cancer are known [9]. Atypical vascular lesions associated with radiation are in a state of continuous transformation, which can progress to more aggressive malignant angiosarcoma [10].

Sarcoma associated with radiotherapy is a rare and aggressive neoplasm that represents between 0.5% and 5.5% of all sarcomas. The prevalence of basal cell carcinoma induced by ionizing radiation is estimated at 4% [11]. Several studies have shown that radiotherapy is a significant risk factor for the development of soft tissue tumors and, in particular, angiosarcomas, after breast cancer treatment [12, 13]. Blanchard *et al.* reported the appearance of sarcomas associated with radiotherapy in 34 patients who received treatment for breast cancer between 1975 and 2001, and 65% of these patients died [14]. Taghian *et al*. reported an accumulated risk of developing soft tissue or bone sarcomas of 0.2% at 10 years within their breast cancer post-treatment cohort of women [15] (see Table 4).

**Table 4:** Review of the literature

Study, year [reference]	Total patients with breast cancer diagnosis	Irradiation dose	Total patients with radiologically induced sarcoma diagnosis (Cahan)	Latency of onset of sarcoma	Location of sarcoma (n)	Type of sarcoma (n)	Mortality due to sarcoma
Blanchard *et al.*, 2002 [14]	251 Mayo Clinic (USA), 1975–2001	50–60 Gy	34	3.3–31 years	ND	Angiosarcoma (12) Malignant fibrous histiocytoma (11) Osteogenic sarcoma (5) Fibroblastic sarcoma (4) Others (2)	22 (65%)
Kirova *et al*., 2005 [16]	16,705 13,472 RT 3233 without radiotherapy (France), 1981–1997	50–55 Gy	27	3–20.3 years	Breast (13) Chest wall (5) Sternum (3) Supraclavicular (2) Scapula (1) Axilla (3)	Angiosarcoma (13) Osteosarcoma (3) Undifferentiated sarcoma (5) Malignant histiocytoma (1) Leiomyosarcoma (2) Fibrosarcoma (1) Rhabdomyosarcoma (1) Myosarcoma (1)	15 (55.5%)
Mery *et al*., 2009 [5]	563,155 211,027 RT, SEER Registry (USA), 1973–2003	ND	146	1–29 years	Chest or ipsilateral upper limb	Angiosarcoma (77) Malignant fibrous histiocytoma (16) Leiomyosarcoma (7) Fibrosarcoma (7) Others (39)	OS 38%
Salminen *et al*., 2018 [17]	132,512 Finnish Registry, 1953–2014	28–60 Gy	96	0.6–29.9 years	Breast (46) Trunk (16) Scar (11) Shoulder (6) Sternum (5) Axilla (4) Lung (4) Scapula (3) Upper Limb (1)	Angiosarcoma (50) Undifferentiated pleomorphic sarcoma (27) Osteosarcoma (5) Fibrosarcoma (3) Extraskeletal osteosarcoma (3) Chondrosarcoma (2) Leiomyosarcoma (2) Myxofibrosarcoma (2) Extraskeletal Chondrosarcoma (1) Neurofibrosarcoma (1)	40 (41.6%)

ND: No Date, OS: Overall survival, RT: radiotherapy

In a study carried out in the radiation therapy oncology department at the Curie Institute in Paris, France, in 2004, where the records of 16,705 patients with breast carcinoma were reviewed, 27 sarcomas associated with radiotherapy were found. The time interval between irradiation and the presence of a second tumor in the irradiated area ranged from 3 to 20.3 years, and the mortality was 55.5% [16]. At the Helsinki Comprehensive Cancer Center, the Finnish Cancer Registry reported a total of 96 patients diagnosed with sarcoma after treatment for breast cancer between 1953 and 2014, with an average latency period of 11.0 (range 0.6–29.9) years [17].

In our institution, 6850 patients treated for breast cancer were found from 2008 to 2018. The time elapsed between diagnosis of the primary tumor and diagnosis of the sarcoma associated with radiotherapy varied between 5 years and 10 years. In the study carried out in Tunisia in 2013, the mean latency for basal cell carcinomas between irradiation and carcinoma was 35.7 years [11]; a longer latency period was found in our case. Atypical radiologically induced vascular lesions occur approximately 3 years after initial treatment [10]. The initial approach begins with physical examination and imaging, but the gold standard is confirmation of the lesion through a biopsy that will corroborate a histological type of tumor different from the primary cancer, as described by Cahan and Arlen [18].

Among different treatments for breast cancer is hypofractionated radiotherapy, which gives a total radiation dose of 40 to 42.56 Gy, but this is not the standard for advanced disease and was not given to any of the seven patients in this series. When comparing hypofractionated radiotherapy with the standard fractioning, they are biologically equivalent, and there is no evidence that different radiotherapy fractioning could increase the risk of second tumors.

The optimal treatment is a wide surgical resection with negative margins, which may result in 5-year survival rates of 40% [19]. However, recurrence and metastases are common, so chemotherapy has become a main component of treatment for sarcomas associated with radiotherapy [20]. Sometimes the benefit of radiotherapy must be established within this therapeutic approach (depending on the histological grade and size of the lesion). The five cases with diagnosis of radiotherapy-induced sarcomas underwent extensive local resection; the patient with well-differentiated vascular lesion was followed in the outpatient clinic; and the patient who had basal cell carcinomas was treated with cryosurgery, as recommended by current literature [21].

Patients with radiation-associated sarcomas have a worse prognosis than patients with primary soft tissue sarcomas; hence, their treatment must be aggressive, including chest wall resections if necessary. Due to the low prevalence of tumors associated with breast cancer radiotherapy, it is difficult to establish a specific monitoring schedule. However, this review aims to provide context for health professionals with regard to the risk of its long-term development and its aggressive behavior and to emphasize the need for timely treatment by a professional who is expert in soft tissue tumors and preferably at a cancer reference center.

Another important aspect to highlight is that, although this entity has a poor prognosis, its low incidence should not affect the decision to administer radiotherapy to patients with breast cancer who have the indication to receive it, because the benefit of radiation therapy is higher than the risk of radiotherapy-induced tumors.

New scientific studies are expected to better define the factors associated with adjuvant radiotherapy that increase the risk of developing other tumors, and to determine whether changes in these factors (for example, smaller radiation fields) would have an impact on the future incidence of the disease.

## Conclusions

Tumors associated with radiotherapy are a low-incidence entity but with high aggressiveness from the local and systemic points of view, which results in high mortality rates. It is noteworthy that radiotherapy is an indispensable therapeutic tool in the treatment of breast cancer, and the presented risk does not outweigh its benefit.

These tumors must be differentiated from local breast cancer relapses, so a biopsy for histological confirmation and a multidisciplinary approach at a reference cancer center are always required. It is expected that new scientific studies will help determine the risk factors for developing this disease and the impact that new technologies will have on the appearance of tumors associated with radiotherapy.

## Data Availability

Data are available in the digital medical records at the National Cancer Institute, Bogota, Colombia. These records are not public, owing to the confidentiality of the type of documents, but they are available from the corresponding author on reasonable request.
